# Light and prey influence the abundances of two rhodopsins in the dinoflagellate *Oxyrrhis marina*

**DOI:** 10.1007/s00709-022-01795-6

**Published:** 2022-07-23

**Authors:** Martin Westermann, Christian Hoischen, Lars Wöhlbrand, Ralf Rabus, Erhard Rhiel

**Affiliations:** 1 Electron Microscopy Center of the Jena University Hospital, Friedrich-Schiller-University Jena, Ziegelmühlenweg 1, 07743 Jena, Germany; 2 CF Imaging, Leibniz Institute On Aging, Fritz–Lipmann–Institute (FLI), Beutenbergstraße 11, 07745 Jena, Germany; 3 General and Molecular Microbiology, Institute for Chemistry and Biology of the Marine Environment, Carl Von Ossietzky University Oldenburg, P.O.B. 2503, 26129 Oldenburg, Germany; 4 Plankton Ecology, Institute for Chemistry and Biology of the Marine Environment, Carl Von Ossietzky University Oldenburg, P.O.B. 2503, 26129 Oldenburg, Germany

**Keywords:** Dinoflagellate, *Oxyrrhis marina*, Immunolabeling, MS analysis, Rhodopsins, Western immunoblotting

## Abstract

Antisera were raised against the C-terminal amino acid sequences of the two rhodopsins ADY17806 and AEA49880 of *Oxyrrhis marina*. The antisera and affinity-purified antibodies thereof were used in western immunoblotting experiments of total cell protein fractions from cultures grown either in darkness or in white, red, green, or blue light. Furthermore, the rhodopsin abundances were profiled in cultures fed with yeast or the prasinophyte *Pyramimonas grossii*. The immunosignals of ADY17806 and AEA49880 were similar when *O. marina* was grown in white, green, or blue light. Signal intensities were lower under conditions of red light and lowest in darkness. Higher amounts were registered for both rhodopsins when *O. marina* was fed with yeast compared to *P. grossii*. Furthermore, total cell protein of cultures of *O. marina* grown under all cultivation conditions was separated by sodium dodecyl sulfate polyacrylamide gel electrophoresis, followed by tryptic in-gel digestion and mass spectrometric analysis of the 25-kDa protein bands. The rhodopsin ADY17809 was detected in all samples of the light quality experiments and in 14 of the 16 samples of the prey quality experiments. The rhodopsin ABV22427 was not detected in one sample of the light quality experiments. It was detected in 15 of the 16 samples of the prey quality experiments. Peptide fragments of the other rhodopsins were detected less often, and no clear distribution pattern was evident with respect to the applied light quality or offered prey, indicating that none of them was exclusively formed under a distinct light regime or when feeding on yeast or the prasinophyte. Fluorescence light microscopy using the affinity-purified antibodies revealed significant labeling of the cell periphery and cell internal structures, which resembled vacuoles, tiny vesicles, and rather compact structures. Immunolabeling electron microscopy strengthened these results and showed that the cytoplasmic membrane, putative lysosome membranes, membranes encircling the food vacuole, and birefringent bodies became labeled.

**Supplementary Information:**

The online version contains supplementary material available at 10.1007/s00709-022-01795-6.

## Introduction

Microbial rhodopsins are photoactive membrane proteins, which are found in archaea, bacteria, and eukaryotic microorganisms. They are characterized by seven membrane-spanning helices labeled A to G, and a retinal chromophore, which is covalently bound at a conserved lysine residue near the C-terminus in helix G. Microbial rhodopsins often form oligomers, i.e., pentamers and hexamers (Klyszejko et al. 2008), and assemblies of higher order, such as the purple membrane of *Halobacterium salinarum*, which is composed of bacteriorhodopsin (Oesterhelt and Stoeckenius 1971). Govorunova et al. (2017) affiliated 12 different types of microbial rhodopsins within the microbial rhodopsin superfamily (see Fig. 1 in Govorunova et al. 2017), but meanwhile, more types such as heliorhodopsins and schizorhodopsins have been discovered (Pushkarev et al. 2018; Inoue et al. 2020). Microbial rhodopsins can be functionally classified according to their involvement in bioenergetics or light sensing. Bioenergetic microbial rhodopsins convert the absorbed light energy into an electrochemical potential, which, in turn, energizes the cell. Light-driven ion pumps that catalyze an active transport of protons (outward-directed), chloride ions (inward-directed), or sodium ions (outward-directed) belong to this group. Photosensory microbial rhodopsins perceive environmental light conditions to appropriately regulate/adapt corresponding cellular processes, such as photomotility. Upon illumination, they either interact with membrane-embedded or soluble transducer proteins or show an enzymatic activity that is encoded in their cytoplasmic domain or induce a signaling cascade as passive light–gated ion channels (see Fig. 2 in Govorunova et al. 2017).

**Fig. 1:**
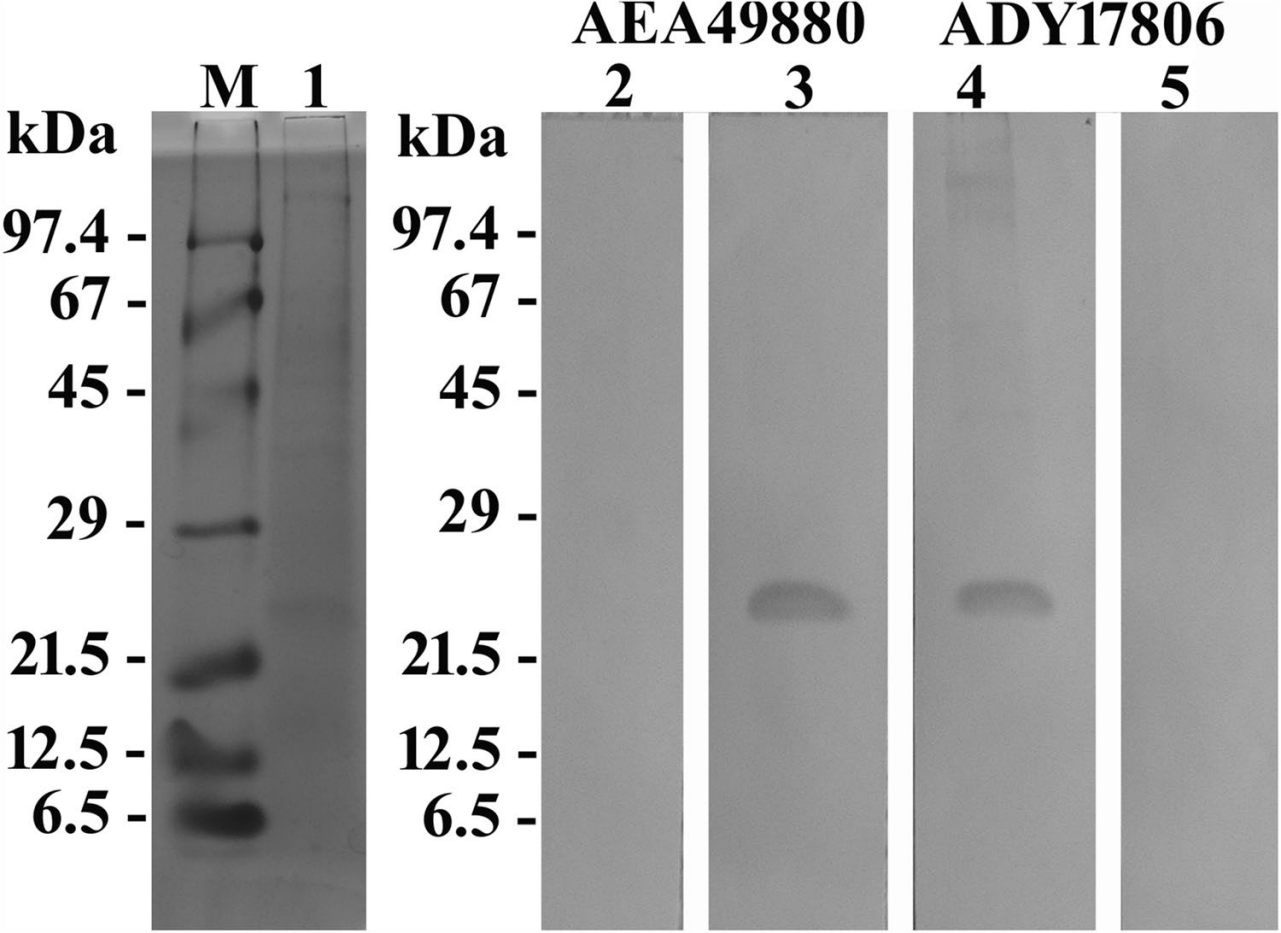
SDS-PAGE and western immunoblots of total cell protein extracts of *O. marina*. Lane M represents the marker, while lanes 1–5 represent the total protein extracts subjected to different detection methods: lane 1, staining with Coomassie; lanes 2–5, western blotting and immunodecoration; lane 2, preimmune serum against AEA49880; lane 3, antiserum against AEA49880; lane 4, antiserum against ADY17806; lane 5, preimmune serum against ADY17806. For better display, the digitized pictures were adjusted for brightness and contrast

**Fig. 2:**
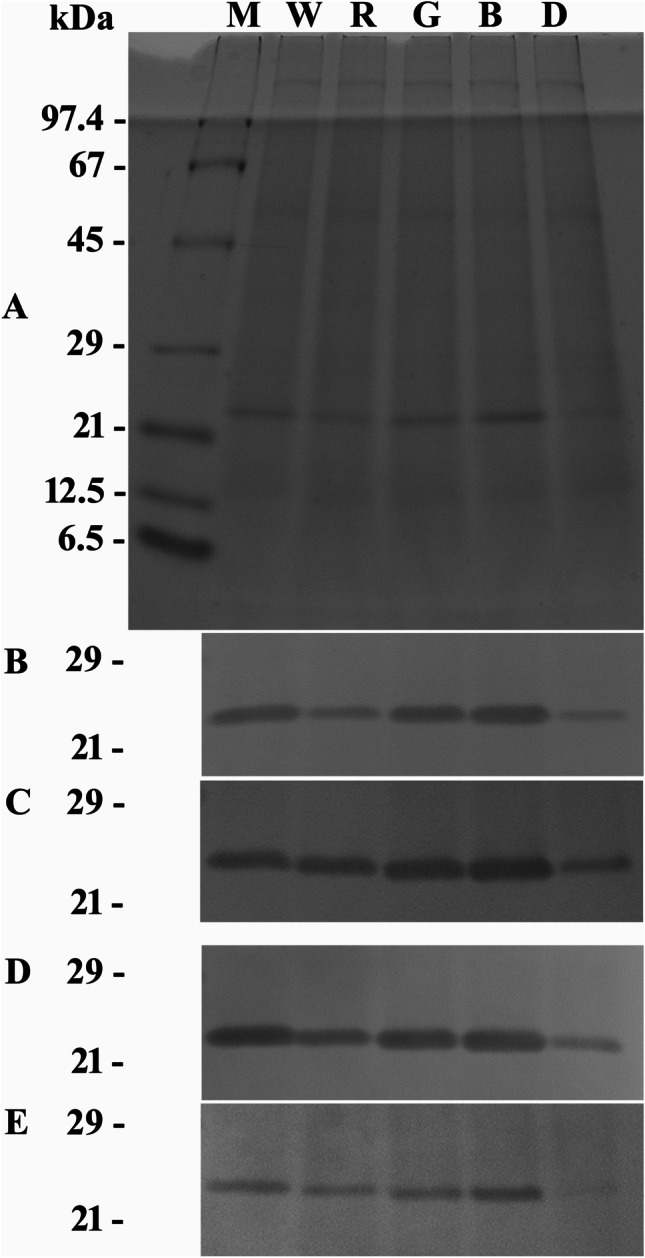
SDS-PAGE-separated total protein extracts of *O. marina* grown under different light regimes. Abbreviations of applied light regimes: W, white; R, red; G, green; B, blue; D, dark. Detection methods applied: Coomassie staining (subfigure **A**; displaying also the size marker, lane M) versus western blotting and immunodecoration with the antiserum against ADY17806 (subfigure **B**) or AEA49880 (subfigure **D**) or the affinity-purified antibodies against ADY17806 (subfigure **C**) or AEA49880 (subfigure **E**). For better display, the digitized pictures were adjusted for brightness and contrast

Proteorhodopsins constitute a highly abundant and widely distributed class of microbial rhodopsins. They function as proton pumps and have been recognized by sequence homology. First discovered by Béjà et al. (2000) in the form of a genomic fragment from uncultured marine γ-proteobacteria, collected in Monterey Bay (CA, USA), proteorhodopsins have meanwhile been described for several bacteria, archaea, and eukaryotic marine protists. With respect to dinoflagellates, (proteo)rhodopsins have been described for *Alexandrium catenella*, *Polarella glacialis*, *Prorocentrum donghaiense*, *Prorocentrum minimum*, *Pyrocystis lunula*, and *Oxyrrhis marina* (Lin et al. 2010; Slamovits et al. 2011; Guo et al. 2014; Shi et al. 2015; and references cited therein). Guo et al. (2014) showed not only the phylogenetic separation but also the distinction of expression response to starvation-light versus starvation-dark. Shi et al. (2015) clustered dinoflagellate proton pump rhodopsin with the group of xanthorhodopsin. Based on sequence analyses, Slamovits et al. (2011) revealed that not all *Oxyrrhis* rhodopsins are proteorhodopsins, as some of them clustered with poorly characterized fungal and algal rhodopsins of unknown functions. Thereafter, however, this distinction became overlooked and the term “proteorhodopsins” was associated with all *Oxyrrhis* rhodopsins. In the present study, the term “rhodopsin” will exclusively be used for those of *Oxyrrhis*. Currently, 19 amino acid sequences of rhodopsins of *O. marina* have been deposited in the NCBI databases (Zhang et al. 2007; Slamovits et al. 2011; Guo et al. 2014). They were deduced from expressed sequence tags (ESTs) and are compiled in Table 1 of Rhiel et al. (2020a). The authors isolated rhodopsin-enriched fractions and verified seven predicted rhodopsin protein sequences experimentally by tryptic in-gel digestion followed by mass spectrometry (Rhiel et al. 2020a). The rhodopsins of *O. marina* are characterized by several conserved functional residues. In OM27 (equivalent to ADY17806) for example, D_101_ and E_112_ in helix C are predicted to function as proton acceptor and proton donor, respectively, K_237_ in helix G is predicted to be linked to the retinal, and L_109_ is assumed to be equivalent to L_105_ in eBAC31A08 that has been shown to be responsible for spectral tuning, i.e., for functioning as a green light absorption tuning switch residue (Man et al. 2003; Slamovits et al. 2011).

**Table 1:** Compilation of the results obtained in the light quality experiments

Light	ADY17806					AEA49880				
	WL	RL	GL	BL	Dark	WL	RL	GL	BL	Dark
Experiments using the antisera										
RGB #9	55,600 (100)	23,200 (41.7)	48,800 (87.8)	57,400 (103.2)	14,200 (25.5)	19,000 (100)	11,200 (58.9)	17,200 (90.5)	17,900 (94.2)	6090 (32.1)
RGB #10	75,900 (100)	47,100 (62.1)	58,100 (76.5)	65,600 (86.4)	16,200 (21.3)	24,100 (100)	18,200 (75.5)	20,000 (83.0)	23,300 (96.7)	10,000 (41.5)
RGB #11	43,600 (100)	25,800 (59.2)	42,000 (96.3)	45,700 (104.8)	12,900 (29.6)	16,500 (100)	8970 (54.4)	11,900 (72.1)	14,400 (87.3)	6560 (39.8)
Experiments using the affinity-purified antibodies										
RGB #9	31,900 (100)	28,400 (90.7)	36,100 (115.3)	46,000 (147)	15,600 (49.8)	26,800 (100)	16,200 (60.4)	23,000 (85.8)	30,800 (114.9)	6260 (23.4)
RGB #10	57,200 (100)	42,900 (75)	64,000 (111.9)	65,600 (114.7)	25,200 (44.1)	53,200 (100)	32,700 (61.5)	41,800 (78.6)	44,800 (84.2)	11,100 (20.9)
RGB #11	96,600 (100)	63,300 (65.5)	91,000 (94.2)	104,000 (107.7)	52,200 (54.0)	20,700 (100)	13,500 (65.2)	31,500 (152.2)	34,600 (167.1)	6340 (30.6)

Equal amounts of total cell protein were subjected to SDS-PAGE, followed by western immunoblotting either using the antiserum against rhodopsin ADY17806 or rhodopsin AEA49880 (upper part: experiments using the antisera) or using the affinity-purified antibodies (lower part: experiments using the affinity-purified antibodies). The values of the measured signal intensities are given. The values in brackets represent the relative amounts (in %) using the values of white light in the light quality experiments (RGB #9–RGB #11) as reference (i.e., set to 100%), respectively

*BL* blue light, *GL* green light, *RL* red light, *WL* white light

Studies on the expression of rhodopsins of dinoflagellates are spare. Guo et al. (2014) investigated the rhodopsin gene expression, i.e., the transcript abundances of *Oxyrrhis* cultures kept either under a light:dark cycle or in continuous darkness. Shi et al. (2015) investigated how light influenced the expression of a rhodopsin in the dinoflagellate *P. donghaiense*. The authors measured both, transcript and protein abundances. For the latter, they used an affinity-purified antibody directed against the C-terminus of the rhodopsin and quantified the signal intensities obtained in western immunoblotting experiments. Investigations on the abundances of rhodopsins of *O. marina* are restricted to the study of Rhiel and Ammermann (2021). The authors used growth parameters, i.e., the final cell densities, the total protein concentrations of the harvested cells, and the 520 nm:740 nm absorbance ratios, or the Coomassie staining intensities of the 25-kDa protein band as measures for the abundances of rhodopsins in response to light and prey quality. Western immunoblotting experiments using antisera/affinity-purified antibodies against distinct rhodopsins of *O. marina* have not been conducted until today. An additional open question is whether or not distinct rhodopsins become exclusively translated under defined light regimes or feeding conditions in *Oxyrrhis*. Meanwhile, two antisera have been raised against two rhodopsins. They were used in immuno-transmission electron microscopy studies, showing that at least these two rhodopsins constitute the birefringent bodies of *O. marina* (Rhiel et al. 2022).

The present study draws on the results of Rhiel and Ammermann (2021) and focuses on the following topics. First, we used the two antisera and affinity-purified antibodies thereof, which were raised in rabbits against the C-terminal amino acid sequences of two rhodopsins of *O. marina*, namely ADY17806 (OM27, Slamovits et al. 2011) and AEA49880, to quantify their abundances under different cultivation conditions. In one set of experiments, *O. marina* was grown in darkness, in white light, or in different light qualities. In another set of experiments, cultures were fed with the prasinophyte *Pyramimonas grossii* instead of dried yeast cells. Total cell protein extracted from *O. marina* cultivated under all of these cultivation conditions was subjected to separation by means of sodium dodecyl sulfate polyacrylamide gel electrophoresis (SDS-PAGE). Then, western immunoblotting using the two generated antisera and the affinity-purified antibodies thereof was conducted in order to quantify the amounts of the two targeted rhodopsins in response to light and prey quality. Furthermore, the diagnostic 25-kDa protein bands were excised from additional SDS-PAGE gels, subjected to tryptic in-gel digestion followed by mass spectrometric analysis to explore if specific formation of distinct rhodopsins in response to the light or feeding regime occurs. Finally, the affinity-purified antibodies were used for immunofluorescence light microscopy and immunolabeling electron microscopy in order to confirm and expand the results obtained earlier by immuno-transmission electron microscopy (Rhiel et al. 2022).

## Material and methods

### Strain sources and growth conditions of stock cultures

*O. marina* was obtained from the Culture Collection of Algae at Göttingen University (strain B21.89; SAG, University of Göttingen, Germany). The prasinophyte *P. grossii* (strain no. K-0253) was obtained from the Norwegian Culture Collection of Algae (NORCCA), which is maintained and owned by the Norwegian Institute for Water Research (NIVA) and the University of Oslo (UiO), Norway. Stock cultures of both organisms were grown in f/2 medium (Guillard and Ryther 1962) at 17 °C without aeration in Erlenmeyer flasks containing 50–1000 ml culture volume. Stock cultures of *O. marina* were fed with dried yeast cells obtained from a local grocery store. Transfer into fresh medium occurred in intervals of 2 weeks. The photon flux density was adjusted to 3 µmol photons m^−2^ s^−1^ using an Almemo 2290–2 measuring instrument (equipped with a FLA613-PSM sensor; Ahlborn Mess- und Regelungstechnik GmbH, Holzkirchen, Germany). The light/dark regime was 14 h:10 h.

### Light and prey quality experiments

The experiments followed the protocols outlined in Rhiel and Ammermann (2021). For the light quality experiments, 250–300 ml cultures of *O. marina*, which were grown with dried yeast cells for 7 days, were mixed with 800 ml fresh f/2 medium. Then, each culture broth was split up into 5 aliquots of 200 ml. These aliquots were then transferred into 250-ml Erlenmeyer flasks, supplemented with 200 µl dried yeast each, and incubated at 17 °C without aeration for 7–8 days either in the dark or in white light or in blue, green, or red light. The different light qualities were achieved using color foils (Rhiel and Ammermann 2021). The photon flux density was adjusted to 8 µmol photons m^−2^ s^−1^, and the light/dark regime was 14 h:10 h. At the end of incubation, the cells were harvested by centrifugation (900 × *g*, 15 min, 18 °C; Eppendorf 5810R refrigerated centrifuge equipped with an A-4–62 swinging bucket rotor; Eppendorf, Hamburg, Germany) and resuspended in 500–600 µl f/2 medium. The concentrated cell suspensions were transferred into 1.5-ml Eppendorf reaction caps and centrifuged (5000 rpm, 10 min, room temperature; Heraeus Fresco bench top micro-centrifuge, Waltham, MA, USA). The resultant cell pellets were suspended in 400 µl of distilled water each and either used immediately, for measuring the protein concentrations and for SDS-PAGE (see “SDS-PAGE and western immunoblotting”), or kept frozen until use. The light quality experiments were repeated three times.

For the prey quality experiments, *O. marina* was fed with dried yeast cells or with *P. grossii* as described earlier (Ammermann et al. 2017; Heyerhoff et al. 2019). The cultures of *O. marina* were harvested 8–14 days after adding the prey organisms. Harvesting of the cells was achieved as described previously. Finally, the cell pellets were resuspended in 400 µl of distilled water each and either used immediately, for measuring the protein concentrations and for SDS-PAGE (see “SDS-PAGE and western immunoblotting”), or kept frozen until use. The prey quality experiments were repeated eight times.

### Antiserum production and affinity purification of antibodies

The antisera directed against the C-termini of rhodopsins ADY17806 and AEA49880 of *O. marina* were obtained from a commercial provider (BioScience, Göttingen, Germany). Synthetic oligopeptides of these two rhodopsins were generated: NAKSRLEEEGKLRA for ADY17806 (according to Slamovits et al. 2011) and NDDLLHVAMPTGVVEQ for AEA49880 (Slamovits and Keeling 2010, direct submission to GenBank, see entry HQ654769.1 for further details). The peptides (2 mg each) were linked to keyhole limpet hemocyanin (KLH) prior to the immunization of rabbits. Preimmune sera were taken before the first immunizations. The specificities of the antisera were tested by western immunoblotting (see “SDS-PAGE and western immunoblotting”).

Affinity-purified antibodies were obtained as follows: 10 mg of the synthetic oligopeptides (see above) were dissolved in 2 ml Tris-buffered saline (TBS, 10 mM Tris–HCl (pH 7.5), 150 mM NaCl) and allowed to absorb onto stripes of Protran BA85 nitrocellulose transfer membrane (pore size 0.45 µm; Schleicher & Schuell GmbH, Dassel, Germany). The stripes were 6 cm in length and 1 cm in width. Then, the stripes were washed with TBS and incubated in TBS containing 1% bovine serum albumin (BSA) in order to block any unspecific binding of antibodies. Two milliliters of the two antisera (see above) was diluted with 8 ml TBS and added to the stripes to which the corresponding oligopeptide has been bound. Binding of corresponding antibodies to the bound oligopeptides was allowed to take place over night. Afterwards, the stripes were exhaustedly washed with TBS. The release of the antibodies was achieved by incubating the stripes for 10 min in 2 ml of 100 mM Tris–HCl (pH 2.5). The solutions were immediately neutralized by the addition of 1 M Tris–HCl (pH 7.5) and used for the immunodecoration of western blots, for immunofluorescence light microscopy, and for immunolabeling electron microscopy (see “Immunofluorescence light microscopy and immunolabeling electron microscopy”).

### SDS-PAGE and western immunoblotting

SDS-PAGE of total protein of *O. marina* cultivated under different light and feeding regimes was performed as described by Bathke et al. (1999). Total protein concentrations were determined from 40-µl aliquots of the concentrated cells according to Lowry et al. (1951) using BSA as standard. The concentrated cells were adjusted to protein concentrations of 1 mg/ml with distilled water and mixed with loading buffer in a 1:1 ratio. Aliquots of 100 µl (equivalent to 50 µg) were heated for 5 min at 95 °C and loaded onto 15% polyacrylamide gels using the buffer system of Laemmli (1970). The loading buffer contained either 5% (v/v) β-mercaptoethanol (β-ME) or 50 mM dithiothreitol (DTT). The gels were run at a constant voltage of 20 V overnight and afterwards stained with Serva Blue R (0.5%, w/v) dissolved in distilled water/methanol/acetic acid (5:5:1), or used for western blotting (see “Quantification of the signals obtained by western immunoblotting”).

The specificities of the antisera were controlled by subjecting total cell protein samples to SDS-PAGE followed by western immunoblotting onto Protran BA85 nitrocellulose transfer membrane according to Towbin et al. (1979). The total cell protein samples obtained in the light and prey quality experiments were also subjected to western immunoblotting. Preimmune sera and antisera (final bleedings) were used in dilutions of 1:2000 (for ADY17806) or 1:500 (for AEA49880). The affinity-purified antibody solutions (see above) were added to 100 ml TBS containing 1% BSA prior to use. Coomassie-stained gels and western immunoblots were digitized using an Olympus C3030 Zoom digital still CCD camera (Olympus, Hamburg, Germany).

### Quantification of the signals obtained by western immunoblotting

The signal intensities of the 25-kDa protein bands detected by western immunoblotting were quantified from digitized images using the Image Studio Lite software (Li-Cor Biotechnology GmbH, Bad Homburg. Germany).

### MS analyses and protein identification

Proteomic analysis was conducted using the rhodopsin-containing 25-kDa protein bands resolved by SDS-PAGE. These were excised from Coomassie-stained gels, washed, and tryptically digested as described by Wöhlbrand et al. (2016). Generated peptides were separated via nanoLC (UltiMate 3000 RSLCnano; Thermo Fisher Scientific, Germering, Germany) and analyzed by online coupling to electrospray ionization (CaptiveSpray ion source; Bruker Daltonik GmbH, Bremen, Germany) and mass analysis by a 3D ion trap (amaZon speed ETD; Bruker Daltonik GmbH) operated as described (Wöhlbrand et al. 2016). Protein identification was performed via the ProteinScape platform (version 3.1, Bruker Daltonik GmbH) on a Mascot server (version 2.3; Matrix Science, London, UK) against available rhodopsin sequences of *O. marina* also including translated EST data (obtained from NCBI, February 2016) and a target-decoy strategy applying described parameters (Wöhlbrand et al. 2016).

### Airyscan super resolution immunofluorescence light microscopy of cells of *O. marina*

The immunofluorescence experiments followed the procedures outlined by Rhiel et al. (2020b). *Oxyrrhis* cells from 100–150 ml culture volume were harvested by centrifugation at 900 × *g* for 10 min in an Eppendorf 5810R refrigerated centrifuge equipped with an A-4–62 swinging bucket rotor (Eppendorf, Hamburg, Germany), and resuspended in 1 ml f/2 medium. Fixation was achieved by adding an equal volume of 8% (v/v) formaldehyde dissolved in f/2 medium to a final concentration of 4%. After an incubation time of 1 h at room temperature, the cells were washed in 20 mM Tris–HCl (pH 7.5) and 150 mM NaCl (TBS) twice for 5 min each. Permeabilization of the cells was achieved by incubating them for 30 min in TBS containing 0.1% (v/v) Triton X-100. After two washings with TBS, the cells were incubated for 2 h at room temperature in 2% (w/v) of BSA dissolved in TBS. Then, the cells were either kept overnight at 4 °C in 2% BSA dissolved in TBS (negative control) or incubated with the undiluted affinity-purified antibody solutions obtained for ADY17806 and AEA49880 (see above). Afterwards, the cells were washed twice with TBS and further incubated for 1 h at room temperature in Alexa Fluor 488–labeled goat anti-rabbit IgG (H + L) conjugate (Invitrogen, Thermo Fisher Scientific, Waltham, USA) diluted 1:1000 in TBS containing 2% BSA. Then, the cells were washed twice in TBS. Fifty microliters of the antisera in 1 ml TBS-resuspended cells was applied to the surface of polylysine-coated microscopy slides (Gerhard Menzel GmbH, Braunschweig, Germany) and incubated for 20 min to allow the cells to settle down and to bind to the microscopy slides. Subsequently, the resuspension was carefully removed and the remaining cells were covered with a cover glass (No. 1.5H, thickness 170 ± 5 µm; Paul Marienfeld GmbH & Co. KG, Lauda-Königshofen, Germany) using ProLong Gold Antifade reagent with DAPI (Invitrogen, Eugene, Oregon).

Airyscan images were acquired in the super resolution mode on a Zeiss LSM 880 microscope (Zeiss, Jena, Germany) equipped with an Airyscan detector using a Plan-Apochromat 63 × /1.4 N.A. oil DIC M27 objective. DAPI was excited with a 405-nm diode (0.6%) using main beam splitter (MBS) 405, and the emission was captured through the following settings: secondary beam splitter (SBS) band path (BP) 420–460 + long path (LP) 500 in combination with emission filter BP 420–445 + BP 465–505 (detector gain 964). Alexa Fluor 488 was excited with a 488-nm argon laser line (1.8%) using MBS 488/561/633, and the emission was captured through an SBS LP 460 in combination with emission filter BP 420–480 + BP 495–550 (detector gain 950). Pixel size in Airyscan super resolution mode acquisition was applied automatically in ZEN 2.3 software for both channels, usually resulting in a pixel size of 35 × 35 nm. The pixel dwell was 1.87 µs, and the line average was 2. Z-Stack scans were performed at 0.153-µm intervals fulfilling Nyquist criteria. Tracks were changed after each Z-stack to reduce the number of SBS and emission filter changes and by this the scan time.

### Immunolabeling electron microscopy of cells of *O. marina*

The immunolabeling electron microscopy experiments, i.e. freeze-fracture immunolabeling (FRIL) on freeze-fracture replica and immunolabeling on ultrathin sections obtained from Oxyrrhis cells were conducted as described earlier (Rhiel et al. 2020b, 2022). In order to get better immunolabeling efficiencies in the FRIL experiments, the evaporation sequence was changed from Pt/C to C/Pt (first carbon 15 nm followed by platinum 2 nm) according to Schlörmann et al. (2007). For these immunolabeling experiments, the affinity-purified antibody solutions obtained for ADY17806 and AEA49880 were used in a dilution of 1:25 for both antibodies.

### Statistical analyses of western immunoblotting

The calculated signal intensities of the immunodecorized 25-kDa protein bands were subjected to one-way analysis of variance (ANOVA) in order to test for treatment effects. For this, SigmaPlot (version 11.0; Systat Software GmbH, Erkrath, Germany) was used. The *p* values smaller than 0.05 (*p* < 0.05) were presumed to indicate significance. Additionally, the signal intensities of the immunodecorized 25-kDa protein bands of cultures representing the white light and yeast feeding conditions were used as 100% reference in the light and prey quality experiments, respectively.

## Results

### The antisera

Total protein extracts were separated by SDS-PAGE across the entire size range from < 10 up to > 100 kDa (Fig. 1, lane 1). Western immunoblotting with both antisera labeled a distinct protein band of 25 kDa size (Fig. 1, lanes 3 and 4). The same was registered for the affinity-purified antibodies (not shown). By contrast, no immunoreactions were observed using the two preimmune sera (Fig. 1, lanes 2 and 5). Thus, the antisera and the affinity-purified antibodies thereof were proved highly specific for the two investigated rhodopsins and, therefore, were used in the subsequent experiments.

### Light quality experiments

Coomassie staining of SDS-PAGE gels revealed almost identical protein banding patterns for all tested light and feeding regimes. Nevertheless, some slight differences in staining intensity of some protein bands can be noticed (Fig. 2A). The results obtained for one of the three light quality experiments by western immunoblotting are shown in Fig. 2B–E, and the results from all experiments are compiled in Table 1.

The antiserum against ADY17806 immunodecorized a 25-kDa protein band in total protein fractions of *O. marina* cells subjected to all light treatments. The signal intensities registered for cells cultured under white, green, or blue light were similar, while lower values were calculated for cells grown in the dark (21–30% as compared to white light) or in red light (41–62%) (see Fig. 2B and Table 1, upper part). One-way ANOVA revealed significant differences for the signals obtained for cells grown in white, blue, or green light vs. those grown in darkness (*p* values: 0.006, 0.008, 0.023), but not vs. those grown in red light. With the signal intensities of cells grown in white light being set to 100%, additional significant differences were calculated for cells grown in white, blue, or green light vs. those grown in red light (*p* values: < 0.001, < 0.001, 0.004) and for red light–grown cells vs. those kept in darkness (*p* value: 0.006).

Western immunoblots using the affinity-purified antibody against ADY17806 showed the same tendency, i.e., similar signal intensities for cells cultured under white, green, or blue light (see Fig. 2C and Table 1, lower part). The values registered for cells cultured under red light or kept in darkness were not as low as registered for the antiserum (66–91% and 44–54% as compared to white light). One-way ANOVA revealed no significant differences for the calculated signal intensities. But when the signal intensities of cells grown in white light were set to 100%, significant differences were registered for cells grown in white, blue, or green light vs. those grown in darkness (*p* values: 0.004, < 0.001, 0.002) and for blue light–grown cells vs. those grown in red light (*p* value: 0.007).

The antiserum against AEA49880 also immunodecorized a 25-kDa protein band from all light treatments and yielded similar signal intensities upon exposition to white, green, or blue light (see Fig. 2D and Table 1, upper part). Again, lower values were calculated for cells grown in the dark or in red light (approximately 32–42% and 54–76% of the signal intensity calculated for white light–grown cells). One-way ANOVA revealed significant differences for the signals obtained for cells grown in white light vs. those grown in darkness (*p* values: 0.036). When the signal intensities of cells grown in white light were set to 100%, significant differences were registered for all combinations (*p* values: < 0.001 to 0.034), except for cells grown in blue light vs. those grown in green light (*p* value: 0.178) and for cells grown in white light vs. those grown in blue light (*p* value: 0.244).

Western immunoblots using the affinity-purified antibody against AEA49880 showed similar results as registered for the antiserum (see Fig. 2E and Table 1, lower part). Here, the signal values for cells grown in red, green, or blue light or for those kept in complete darkness were approximately 60–65%, 78–152%, 84–167%, and 21–31% of the values calculated for cells grown in white light (set to 100%). The mean values among the different treatments and when the signal intensities of cells grown in white light were set to 100% showed statistically significant differences (*p* values: 0.039 and 0.025).

### Prey quality experiments

**Fig. 3:**
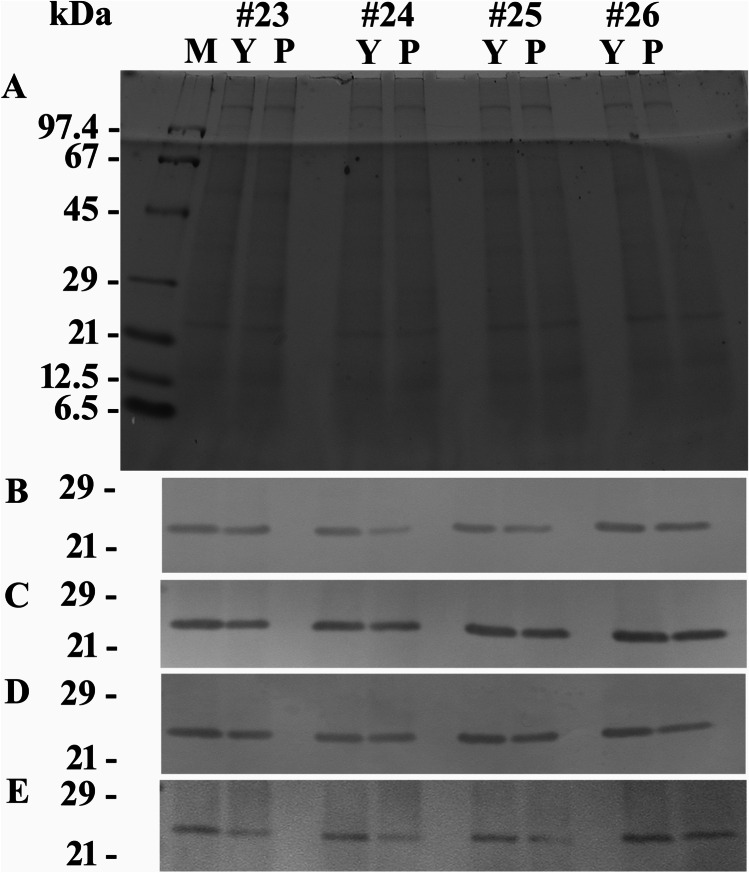
SDS-PAGE-separated total protein extracts of *O. marina* grown under different feeding regimes. Abbreviations of applied feeding: Y, yeast; P, the prasinophyte *Pyramimonas grossii*. Results from four of the eight replicate experiments (#23–#26) are shown. Detection methods applied: Coomassie staining (subfigure **A**; displaying also the size marker, lane M) versus western blotting and immunodecoration with the antiserum against ADY17806 (subfigure **B**) or AEA49880 (subfigure **D**) or the affinity-purified antibodies against ADY17806 (subfigure **C**) or AEA49880 (subfigure **E**). For better display, the digitized pictures were adjusted for brightness and contrast

As registered in the light quality experiments, Coomassie-stained gels showed similar protein banding patterns for cells fed either with yeast or with *P. grossii* (Fig. 3A). The results obtained by western immunoblotting from four of the eight experiments are shown in Fig. 3B and D (using the two antisera) and in Fig. 3C and E (using the affinity-purified antibodies). The calculated signal intensities from all experiments are compiled in Table 2.

**Table 2:** Compilation of the results obtained in the prey quality experiments (#19–#26)

Prey	ADY17806		AEA49880	
	Yeast	*Pyramimonas grossii*	Yeast	*Pyramimonas grossii*
Experiments using the antisera				
#19	43,800 (100)	32,200 (73.5)	31,700 (100)	25,600 (80.8)
#20	54,400 (100)	32,900 (60.5)	28,100 (100)	16,500 (58.7)
#21	34,900 (100)	27,700 (79.4)	29,600 (100)	15,400 (52.0)
#22	36,800 (100)	22,900 (62.2)	26,600 (100)	18,800 (70.7)
#23	35,600 (100)	28,500 (80.1)	49,900 (100)	36,800 (73.8)
#24	30,400 (100)	13,500 (44.4)	35,100 (100)	30,700 (87.5)
#25	27,800 (100)	21,000 (75.5)	47,000 (100)	37,300 (79.4)
#26	41,600 (100)	37,600 (90.4)	53,600 (100)	33,300 (62.1)
Experiments using the affinity-purified antibodies				
#19	38,300 (100)	38,000 (99.2)	7660 (100)	3720 (48.6)
#20	49,600 (100)	28,300 (57.1)	7580 (100)	4710 (62.1)
#21	38,300 (100)	28,900 (75.5)	3260 (100)	4510 (138.3)
#22	33,100 (100)	29,500 (89.1)	3790 (100)	1850 (48.8)
#23	96,900 (100)	60,500 (62.4)	8130 (100)	4210 (51.8)
#24	82,600 (100)	71,200 (86.2)	8160 (100)	3230 (39.6)
#25	93,100 (100)	77,800 (83.6)	8030 (100)	2310 (28.8)
#26	126,000 (100)	109,000 (86.5)	9470 (100)	5370 (56.7)

Equal amounts of total cell protein were subjected to SDS-PAGE, followed by western immunoblotting either using the antiserum against rhodopsin ADY17806 or rhodopsin AEA49880 (upper part: experiments using the antisera) or using the affinity-purified antibodies (lower part: experiments using the affinity-purified antibodies). The values of the measured signal intensities are given. The values in brackets represent the relative amounts (in %) using the values of cells grown with yeast as prey as reference (i.e., set to 100%), respectively

The two antisera immunodecorized 25-kDa protein bands in total protein extracts of *O. marina* fed with yeast or fed with the prasinophyte *P. grossii*, albeit with stronger signal intensities in case of yeast (see Fig. 3B, D and Table 2, experiments using the antisera). For rhodopsin ADY17806, the signal intensities detected under the *P. grossii* regime reached approximately 44–90% of the values calculated for feeding with yeast (Fig. 3B and Table 2, experiments using the antisera). When using the antiserum against AEA49880, the signal intensity of the 25-kDa protein band upon feeding with the prasinophyte accounted for approximately 52–88% of the values registered with yeast (Fig. 3D and Table 2, experiments using the antisera). One-way ANOVA revealed significant differences between the two feeding regimes for both antisera, resulting in *p* values of 0.015 and 0.046 for the immunosignals obtained for ADY17806 and AEA49880, respectively. Even smaller *p* values (< 0.001) were calculated when the signal intensities of cells grown on yeast were set to 100%.

The calculated values of the signal intensities from western immunoblots using the affinity-purified antibodies against ADY17806 and AEA49880 showed the same tendencies as registered for the two antisera (see Fig. 3C, E and Table 2, experiments using the affinity-purified antibodies). However, it was less pronounced for the affinity-purified antibody against ADY17806: 57–99% of the values registered for cells fed with yeast were found for cells fed with the prasinophyte. One-way ANOVA reflects this finding, as no statistically significant difference was registered in this case and when using the measured signal intensities. A *p* value of < 0.001 was calculated when the signal intensities of cells grown on yeast were set to 100%. A statistically significant difference between the two prey treatments with *p* values of 0.004 (when using the measured signal values) and 0.011 (when the signal intensities of cells grown on yeast were set to 100%) was calculated for the affinity-purified antibody against AEA49880. Here, with one exception (experiment #21), the signal intensities registered for cells fed with *Pyramimonas* reached 29–62% of the values of cells fed with yeast (see Table 2, experiments using the affinity-purified antibodies).

### MS analysis and protein identification

A compilation of the results obtained by MS analyses from the light and prey quality experiments is given in Table 3, and the raw data are added in the Supplemental Excel file “MS data of the *Oxyrrhis marina* light quality and prey quality experiments.” In total, nine different rhodopsin sequences were identified by MS analyses in the experiments, namely ADY17806, ADY17808, ADY17809, ADY17811, ABV22426, ABV22427, ABV22430, ABV22432, and AEA49880. Three to four rhodopsins were registered in each of the excised 25-kDa protein bands from the light quality experiments (Table 3, upper part), whereas two to four rhodopsins from the prey quality experiments (Table 3, lower part). The rhodopsin ADY17809 was detected in all samples of the light quality experiments and in 14 of the 16 samples of the prey quality experiments. ABV22430 was also registered for most of the light quality samples and only missed twice for cells grown in red light (experiments RGB #9 and RGB #11). It was detected in only half of the samples of the prey quality experiments. Peptides of ABV22427 were detected in 12 of the 15 samples of the 3 light quality experiments and in 15 of the 16 samples of the prey quality experiments. Peptides of ABV22430 were registered in most of the light qualities, too, and only missed twice for protein samples of cells grown in red light. It was detected in only half of the samples of the prey quality experiments. Peptides of the other rhodopsins were detected less often, and no clear distribution pattern was evident with respect to the applied light quality or offered prey. In addition to rhodopsins, a few peptides of other abundant cellular proteins, such as α-tubulin, β-tubulin, and ribosomal proteins, were detected in the experiments (see Supplemental Excel file).

**Table 3:** Summary of rhodopsin identification by mass spectrometry in the light (upper part) and prey (lower part) quality experiments

	*RGB #9*										*RGB #10*										*RGB #11*									
	WL		RL		GL		BL		D		WL		RL		GL		BL		D		WL		RL		GL		BL		D	
ADY17806.1	+						+										+													
ADY17809.1	+		+		+		+		+		+		+		+		+		+		+		+		+		+		+	
AEA49880.1	+																				+		+		+					
ABV22432.1			+																											
ABV22430.1	+				+		+		+		+		+		+		+		+		+				+		+		+	
ABV22427.1			+		+				+		+		+		+				+		+		+		+		+		+	
ABV22426.1																							+							
	*#19*			*#20*				*#21*				*#22*				*#23*				*#24*				*#25*				*#26*		
	Y	P		Y		P		Y		P		Y		P		Y		P		Y		P		Y		P		Y		P
ADY17806.1																								+						
ADY17808.1						+		+				+																		
ADY17809.1	+			+		+		+		+		+		+		+		+		+				+		+		+		+
ADY17811.1				+																										
AEA49880.1												+																		
ABV22430.1	+			+										+		+				+		+				+		+		+
ABV22427.1	+	+		+		+		+		+		+		+		+		+		+		+				+		+		+
ABV22426.1		+								+								+						+						

Detection of the respective rhodopsin (first column) is indicated by a plus sign; #19–#26 are prey quality experiments; RGB #9–RGB #11 are light quality experiments

*BL* blue light, *D* dark, *GL* green light, *P Pyramimonas grossii* as prey, *RL* red light, *WL* white light, *Y* yeast as prey

### Immunofluorescence light microscopy and immunolabeling electron microscopy

**Fig. 4:**
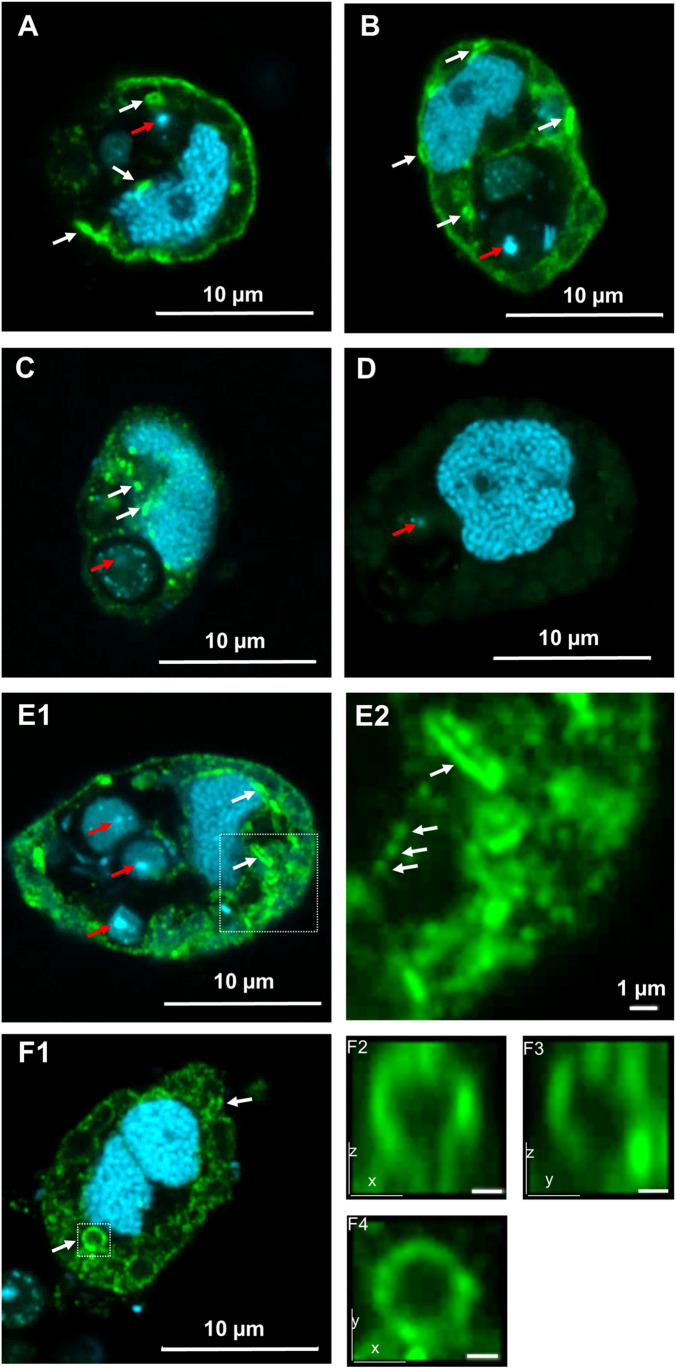
Airyscan super resolution fluorescence microscopical analysis of *O. marina*. The localization of rhodopsin is shown in green (Alexa Fluor 488) after primary detection with the affinity-purified antibody ADY17806 (**A** and **B**), the affinity-purified antibody AEA49880 (**C**), and as a control without primary antibody (**D**). DAPI-stained DNA is shown in light blue. For better evaluation and comparison of the rhodopsin staining for **A**–**D**, the microscopical setup was identical and the presentation of the generated images (brightness, contrast) was performed under exactly the same conditions. An ADY17806-labeled cell exhibiting numerous rhodopsin-labeled membrane structures (green) is shown in **E1**, in which the DNA is shown in light blue. The area marked with the white square is magnified in **E2**, giving a better view of the rhodopsin structures (DNA is not visualized). **F1** ADY17806-labeled cell with rhodopsin located in large membrane vesicle structures (green). DAPI-stained DNA is light blue. The area in the white square containing a large vesicular structure is visualized in more detail in **F2**, **F3**, and **F4** (DNA is not shown). **F2** represents a XZ projection and **F3** a YZ projection of this area, both generated from a 3D reconstruction of the cell using the Z-stack images indicating a bulb-like 3D structure of the rhodopsin-containing membranes in the magnified area. A plain XY magnification is shown in **F4**. Concise rhodopsin-labeled areas of the cytoplasmic membrane, such as vesicle-like structures and birefringent bodies, are marked with white arrows. Red arrows indicate the DNA of incorporated yeasts. Scale bars are indicated, except in **F2**, **F3**, and **F4** where a scale bar represents 0.5 µm. For better contrast, the pixels in micrographs **E2**, **F2**, **F3**, and **F4** have been interpolated

The results obtained by immunofluorescence light microscopy of cells of *O. marina* are compiled in Fig. 4. Using the affinity-purified antibody solution for ADY17806 prior to the second antibody resulted in an intense labeling of several cellular structures. Thus, the cell periphery (Fig. 4A, B, E1, F1), compact stick-like structures representing most likely the birefringent bodies (see structure in the upper right part of the cell shown in Fig. 4B and structure in the upper part of Fig. E2), and membranes encircling vacuoles became labeled (see Fig. 4F1, F2, F3, F4). Minor cellular structures, which resembled tiny vesicles, became labeled, too. The results were similar when the affinity-purified antibody solution obtained for AEA49880 was used (Fig. 4C). The fluorescence intensity, however, was weaker. Using the Alexa Fluor 488–labeled second antibody alone did not give rise to specific and distinct green fluorescence signals at all (Fig. 4D). Quantification of the immunofluorescence with the affinity-purified antibodies was not performed for cells either grown under the different light regimes or fed with yeast or the prasinophyte due to several reasons (see “Western immunoblotting vs. quantitative immunofluorescence with affinity-purified antibodies”).

**Fig. 5:**
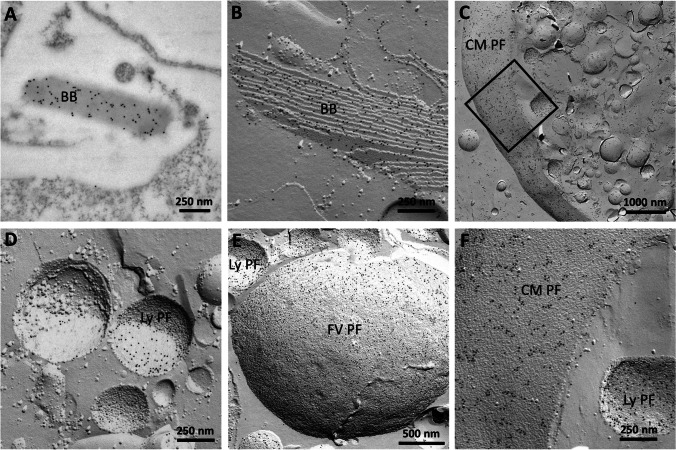
Immunolabeling electron microscopy of an ultrathin section (**A**) shows the labeling of a birefringent body (BB) using the affinity-purified antibody for ADY17806. After FRIL experiments, the membranes of BBs are labeled similarly (**B**). Intense labeling is also present at the protoplasmic fracture faces (PF) of the cytoplasmic membrane (CM PF) (**C**, detail in **F**). The PF of small vesicles with diameters of 200–500 nm (**D**, also in **E** and **F**) are labeled intensely. These vesicles may represent lysosomes (Ly PF). Big vesicular structures with diameters of about 3–5 µm (**E**) are labeled at the PF. These big vesicular structures may represent the membranes encircling the food vacuoles (FV PF) (**E**). Scale bars are indicated

**Fig. 6:**
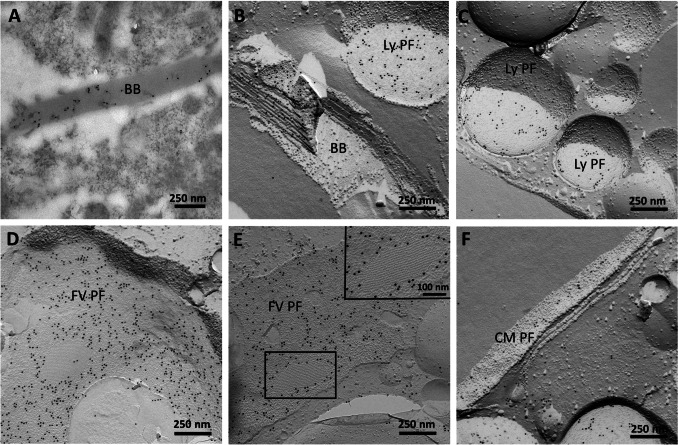
Immunolabeling electron microscopy of an ultrathin section (**A**) shows the labeling of a birefringent body (BB) using the affinity-purified antibody for AEA49880. After FRIL experiments, the membranes of BBs are labeled similarly (**B**). The protoplasmic fracture faces (PF) of small vesicles with diameters of 200–500 nm (**B**, **C**) are labeled intensely. These vesicles may represent lysosomes (Ly PF). Big vesicular structures with diameters of about 3–5 µm (**D**, **E**) are labeled at the PF. These big vesicular structures may represent the membranes encircling the food vacuoles (FV PF) (**D**, **E**). The detail view in **E** shows a membrane imprint of a birefringent body stack. Some label is also present at the PF of the cytoplasmic membrane (CM PF) (**F**). Scale bars are indicated

Immunolabeling electron microscopy on ultrathin sections confirmed the findings of Rhiel et al. (2022). The results are compiled in Figs. 5A and 6A, and showed intense labeling of the birefringent bodies when using the affinity-purified antibody solution obtained for ADY17806 (Fig. 5A) or for AEA49880 (Fig. 6A). FRIL experiments revealed labeling of the birefringent bodies, too (Figs. 5B and 6B). In addition, intense labeling was registered for the protoplasmic fracture faces (PF) of the cytoplasmic membrane (Fig. 5C, detail in Fig. 5F, and Fig. 6F), of small vesicles with diameters of 200–500 nm (Figs. 5D and 6B, C), and of big vesicular structures with diameters of 3–5 µm that may represent the membranes encircling the food vacuoles (Figs. 5E and 6D, E). Based on size and content appearance, the small vesicles most likely correspond to lysosomal vesicles.

## Discussion

### Why choosing the rhodopsins AEA49880 and ADY17806 for the production of antisera

Currently, 19 rhodopsin sequences of *O. marina* are deposited in the NCBI databases. Based on the phylogenetic distribution of dinoflagellate rhodopsins displayed in Fig. 1 of Slamovits et al. (2011) and on the ClustalO sequence alignment of the *O. marina* rhodopsin sequences compiled in Table 2 of Rhiel et al. (2020a), it appears plausible to groups ABV22426, ABV22427, ABV22428, ABV22429, ABV22430, ABV22431, ABV22432, ADY17806, ADY17807, ADY17808, ADY17809, and ADY17811 in the “Dinoflagellate 1” cluster. The latter represents a large group of H^+^-pumping rhodopsins, which are present in diverse dinoflagellates (Slamovits et al. 2011). The rhodopsins AIN36546, AIN36547, AIN36548, AIN36549, AEA49880, and ADY17810 (equivalent to OM2197 in Slamovits et al. 2011) can be allocated to the “Dinoflagellate 3” cluster, which includes genes related to algal sensory rhodopsins. Considering that so far only peptides of ADY17806, ADY17808, ADY17809, ADY17811, ABV22426, ABV22427, ABV22430, and AEA49880 have been detected by MS analyses in rhodopsin-enriched fractions of the *O. marina* strain investigated by Rhiel et al. (2020a), the two antisera were raised against the C-termini of ADY17806 (H^+^-pumping rhodopsin) and AEA49880 (algal sensory rhodopsin), respectively. Noteworthy, the amino acid residues, which are proposed to function in H^+^ translocation, retinal binding, and spectral tuning are conserved in these two proteins. Furthermore, it has to be considered that the C-terminal amino acid sequences of ADY17806 and ABV22427 are identical and, thus, the antiserum against ADY17806 should also recognize ABV22427. This might explain the results obtained by western immunoblotting (see below).

### Light availability and light quality seem to influence the amounts of both rhodopsins

Lowest amounts of the rhodopsins AEA49880 and ADY17806 (and most likely ABV22427 as well) were detected for cells grown in darkness, followed by those grown in red light, while similarly higher values were observed for cells grown either in white or in green or in blue light. This agrees with the results reported by Shi et al. (2015) for *Prorocentrum donghaiense*. The authors measured the transcript and protein abundances of a H^+^-pumping rhodopsin (GenBank accession number: KM282617) by means of reverse transcription quantitative PCR (RT-qPCR) and western immunoblotting using an antiserum directed against its C-terminal amino acid sequence. It turned out that the transcript abundance showed a diel rhythm when the alga was cultured under a light/dark cycle with higher values in the light phase. The transcript abundances were also higher when the alga was grown in white, blue, and green lights, and less when the cultures were kept in red light. The protein was more abundant in the light period and at moderate light intensity, i.e., at 100 µE·m^−2^·s^−1^. Growing the culture in the dark or at a much higher light intensity, i.e., at 200 µE·m^−2^·s^−1^, caused reduced protein abundances.

The results of the present study indicate that distinct rhodopsins of *O. marina*, i.e., AEA49880 and ADY17806/ABV22427, are differently abundant under specific light conditions. This result somewhat contradicts the findings of Rhiel and Ammermann (2021). The authors grew *Oxyrrhis* with yeast cells under the same light regime as used in the present study. However, they did not register significantly differing amounts of rhodopsins using the absorbance ratio 520 nm:740 nm as measure. Significant differences in the finally reached cell densities and total protein concentrations of the harvested cells were also not detected, and no differences were found for the Coomassie staining intensities of the 25-kDa protein band. However, these measures/methods are less sensitive as compared to western immunoblotting experiments using antisera against distinct rhodopsins, which were not conducted in this earlier study.

### Prey quality also seems to influence the amounts of both rhodopsins

Western immunoblotting revealed that total cell protein extracts of *O. marina* gave rise to lower signal intensities for the rhodopsins ADY17806 (and most likely ABV22427 as well) and AEA49880 when the cultures were fed with *P. grossii*. This is in line with the findings of Rhiel and Ammermann (2021), who investigated the influence of prey organisms on the amounts of rhodopsins of *O. marina*. The authors did not detect any rhodopsin-containing bands by sucrose density gradient centrifugation of Triton X-100-solubilized membrane fractions when *P. grossii* was used as prey organism. The 25-kDa protein band was almost absent upon separation of the gradient bands and the non-solubilized pellet by SDS-PAGE coupled to staining with Coomassie. Western immunoblotting experiments were not conducted in this earlier study. Based on the results of the present study, prey quality seems to have an impact on the formation of distinct rhodopsins in *O. marina*.

### Western immunoblotting vs. quantitative immunofluorescence with affinity-purified antibodies

It would have been challenging to compare the results obtained by western immunoblotting with those obtained by immunofluorescence light microscopy. However, the immunofluorescence signals, which we registered for cells belonging to the same treatment with respect to light or prey quality, were not uniform and homogeneous. We therefore discarded the idea to quantify the signal intensities for cells of the light quality and prey quality experiments. To our opinion, (1) the amounts of birefringent bodies, (2) the amounts of preyed cells, and thus (3) the amounts of membranes belonging to the food vacuole within cells grown under the same regime of either light or prey quality experiment varied. This would finally result in measures showing large variances for each of the different treatments, and to make matters worse, the cells tend to aggregate during the preparation, making signal quantification of single cells more difficult. The latter finding most likely was due to discharged trichocysts, which formed net-like structures within which the cells became captured.

### The results of the MS analyses

Peptides of rhodopsin ABV22432 were detected in addition to rhodopsins already described by Rhiel et al. (2020a). ADY17809 seems to be constitutively formed as it was detected in all samples of the light quality experiments and was missing in only two samples of the prey quality experiments. A similar assumption can be made for ABV22427 and ABV22430. Peptides of the other rhodopsins were detected less often, and no clear distribution pattern was evident. Thus, the results obtained by MS analyses revealed no strict correlation between the presence or absence of a distinct rhodopsin and either the applied light regime or the offered prey.

We assume for the two rhodopsins examined in the present study that they might vary with respect to their total amounts under the applied light regimes or added prey, but none of the two seems to be strictly expressed/repressed in a light- or prey-dependent manner. Interestingly, peptides of AEA49880 were detected only four times in the light quality experiments and once in prey quality experiment #22. This is striking as we detected the protein by western immunoblotting in all samples. The same applies to ADY17806 as peptides of this rhodopsin were detected less often. This finding thus seems also to differ from the results obtained by western immunoblotting as the antiserum against the C-terminal amino acid sequence of this rhodopsin nicely worked and indicated rather high amounts of this protein. But as already mentioned, the C-terminal amino acid sequences of ADY17806 and ABV22427 are identical, which might explain the obtained results, in particular as ABV22427 seems to be constitutively formed. As an additional explanation, one might argue that western immunoblotting is much more sensitive than MS analysis. Peptides of eleven of the predicted rhodopsins have not been detected at all by MS analysis in the present study. This finding might have several additional reasons, i.e., (1) the coding genes are not present, (2) they are not formed in the *O. marina* strain investigated, (3) they are present in amounts below the detection limit, or (4) they are not accessible to tryptic digestion or ionization of peptides failed. The last assumption might be true for AEA49880 as well. RT-PCR and antisera against the C-termini of additional rhodopsins of *O. marina* might help to elucidate the formation of this interesting group of proteins in more detail.

### Immunofluorescence light microscopy and immunolabeling electron microscopy of cells of *O. marina*

In a previous study, Rhiel et al. (2020b) used an antiserum against a rhodopsin-enriched fraction. Subsequent immunofluorescence light microscopy studies with the antiserum resulted in intense labeling of the periphery of *Oxyrrhis* cells. Some cell internal structures became also labeled. These results were confirmed in the current study using the affinity-purified antibodies. Compared to the results obtained by using the original antisera, both affinity-purified antibodies showed in light and electron microscopy a higher labeling efficiency and less labeling background. Both affinity-purified antibody solutions labeled the same cellular structures, but the one against AEA49880 resulted in a weaker labeling than the one against ADY17806. This result was not registered by immunolabeling electron microscopy in the current study, as both antibody solutions gave rise to similar labels. Currently, we do not have any explanation for this. In the earlier study (Rhiel et al. 2020b), immunoelectron microscopy of freeze-fractured cells showed that most likely the membranes of the amphiesmal vesicles were labeled at the cell periphery. Labeling of the cytoplasmic membrane could not be confirmed as almost no areas of the cytoplasmic membranes were fractured. The cell internal label seemed to originate from the food vacuoles. Rhiel and Ammermann (2021) failed to use this antiserum in western immunoblotting experiments as several additional protein bands became labeled. The authors suggested that most likely trichocyst fragments contaminated the rhodopsin fraction and caused the unspecific labeling. In the current study, synthetic oligopolypeptides were used as antigens. One might assume that this approach is a backward step. A major advantage using native isolated rhodopsins for immunization surely is that the antiserum will detect several different rhodopsins, and most likely several antigenic protein epitopes within each of the rhodopsins as well. Investigations on the abundance and localization of a distinct rhodopsin, however, cannot be performed with such an antiserum. An additional problem might be that the rhodopsin fraction used for immunization is not absolutely devoid of contaminating proteins, thus finally resulting in a polyspecific antiserum that might cause false-positive results with respect to abundance and cellular localization. Synthetic oligopeptides, which represent the C-terminal amino acid sequences of distinct rhodopsins, do not show these problems. When used as antigens, they finally result in antisera from which the correct antibodies can easily become affinity-purified. Meanwhile, both antisera described in the current study have been used in an immunoelectron microscopy study and it was demonstrated that both rhodopsins, ADY17806 and AEA49880, are localized within the birefringent bodies of *O. marina* (Rhiel et al. 2022). This finding was confirmed in the current study using the affinity-purified antibodies. Additionally, the cytoplasmic membrane, the membranes encircling the food vacuoles, and most likely lysosomal membranes became labeled. The labeling of lysosomal and food vacuole membranes confirms the earlier assumption that rhodopsins most likely acidify the interior of food vacuoles, thus facilitating digestion processes. The finding that the cytoplasmic membrane showed labeling confirms the results of Hartz et al. (2011) and Ma et al. (2020) and might be interpreted a ATP-generating process via proton translocation across that membrane.

## Supplementary Information

Below is the link to the electronic supplementary material.

Supplementary file1 (XLSX 21 KB)
